# Deep Sea Water Modulates Blood Pressure and Exhibits Hypolipidemic Effects via the AMPK-ACC Pathway: An *in Vivo* Study 

**DOI:** 10.3390/md11062183

**Published:** 2013-06-17

**Authors:** Ming-Jyh Sheu, Pei-Yu Chou, Wen-Hsin Lin, Chun-Hsu Pan, Yi-Chung Chien, Yun-Lung Chung, Fon-Chang Liu, Chieh-Hsi Wu

**Affiliations:** 1 School of Pharmacy, China Medical University, 91 Hsueh-Shih Road, Taichung 404, Taiwan; E-Mails: linwh0777@gmail.com (W.-H.L.); joseph.panch@gmail.com (C.-H.P.); hardway19800710@gmail.com (Y.-C.C.); p730912@hotmail.com (Y.-L.C.); 2 Department of Nutrition, China Medical University, 91 Hsueh-Shih Road, Taichung 404, Taiwan; E-Mail: peiyu67@gmail.com; 3 Department of Pharmacy, Da-Chien General Hospital, Miaoli 36052, Taiwan; E-Mail: fonchang008@yahoo.com.tw

**Keywords:** deep sea water, atherosclerosis, HMG-CoA reductase, AMP-activated protein kinase, acetyl-CoA carboxylase

## Abstract

Deep sea water (DSW), originally pumped from the Pacific Rim off the coast of Hualien County (Taiwan), and its mineral constituents, were concentrated by a low-temperature vacuum evaporation system to produce a hardness of approximately 400,000 mg/L of seawater mineral concentrate. The primary composition of this seawater mineral concentrate was ionic magnesium (Mg^2+^), which was approximately 96,000 mg/L. Referring to the human recommended daily allowance (RDA) of magnesium, we diluted the mineral concentrate to three different dosages: 0.1 × DSW (equivalent to 3.75 mg Mg^2+^/kg DSW); 1 × DSW (equivalent to 37.5 mg Mg^2+^/kg DSW); and 2 × DSW (equivalent to 75 mg Mg^2+^/kg DSW). Additionally, a magnesium chloride treatment was conducted for comparison with the DSW supplement. The study indicated that 0.1 × DSW, 1 × DSW and 2 × DSW decreased the systolic and diastolic pressures in spontaneous hypertensive rats in an eight-week experiment. DSW has been shown to reduce serum lipids and prevent atherogenesis in a hypercholesterolemic rabbit model. Our results demonstrated that 1 × DSW and 2 × DSW significantly suppressed the serum cholesterol levels, reduced the lipid accumulation in liver tissues, and limited aortic fatty streaks. These findings indicated that the antiatherogenic effects of DSW are associated with 5′-adenosine monophosphate-activated protein kinase (AMPK) stimulation and the consequent inhibition of phosphorylation of acetyl-CoA carboxylase (ACC) in atherosclerotic rabbits. We hypothesize that DSW could potentially be used as drinking water because it modulates blood pressure, reduces lipids, and prevents atherogenesis.

## 1. Introduction

Approximately 50% of all deaths from cardiovascular diseases (CVD) in Western countries are due to coronary heart disease (CHD), and the primary cause of CHD is atherosclerosis. It is hypothesized that atherosclerosis begins when the endothelium becomes damaged, thereby allowing low-density lipoprotein cholesterol (LDL-C) to accumulate on the artery wall. Subsequently, lipid accumulation, foam cell development, and vascular smooth muscle cell proliferation occur, and finally, the arteries become narrowed and hardened [1]. Hypercholesterolemia and high levels of LDL cholesterol are two important stimuli that regulate the pathogenesis of atherosclerosis [1,2]. Steinberg *et al*. demonstrated that oxidized LDL (oxLDL) is a key element involved in atherosclerotic plaque formation and atherogenicity [3]. When oxLDL is uptaken by vascular scavenger receptors, the transformation of macrophages into foam cells is triggered in atherosclerotic lesions [4]. A strong correlation between hypertension and CHD is widely under investigation. Several pathophysiologic mechanisms link both hypertension and CHD. Hypertension induces endothelial dysfunction, exacerbates the atherosclerotic process and contributes to make the atherosclerotic plaque more unstable [5].

An effective and safe drug to hypercholesterolemia would be beneficial for the prevention of atherosclerosis. Clinically, HMG-CoA reductase (HMGCR) inhibitors (*i.e.*, statins) are commonly prescribed for hyperlipidemia. Statins reduce the incidence of CHD by 23%–34% and mortality by 20%–42% [6]. Because there are limited cases of statin-induced rhabdomyolysis, it is essential to develop new and safer lipid-lowering agents.

5′-Adenosine monophosphate-activated protein kinase (AMPK) plays an important role in regulating the different phases of intermediary metabolism, which include glucose transport, gluconeogenesis, glycogenolysis, lipolysis, and sterol synthesis [7,8]. AMPK, a key cellular sensor for energy homeostasis, consists of a catalytic subunit (α) and two regulatory subunits (β, γ), which bind to form a functional kinase [9]. AMPK is activated by metabolic stress or exercise that reduces cellular energy levels, which indicates an increase in the AMP/ATP ratio due to the depletion of ATP. Therefore, AMPK advances the ATP-generating pathway and reduces the ATP-consuming pathway [10]. Under fasting conditions, the activated AMPK regulates several intracellular metabolic systems to generate energy or reduce energy depletion. Predominantly, the metabolic modifications include the acceleration of lipid catabolism via the suppression of ACC [11], the inhibition of cholesterol synthesis via the depressed activity of HMGCR [12], and decreased fatty acid *de novo* biosynthesis via the suppression of fatty acid synthase [13]. For that reason, AMPK protein may be considered to be a pharmacological target for the management of hyperlipidemia or atherosclerosis.

Characterized by its clarity, sanitary quality, plentiful nutrients, particularly rich in ionic magnesium (Mg^2+^), calcium (Ca^2+^), and potassium (K^+^), deep sea water (DSW) has received attention for its potential in various disease treatments, including attenuation of hyperlipidemia, atherosclerosis [14], hypertension [15], and dermatitis syndrome [16]. Recently, DSW has been widely investigated for its therapeutic or preventive effects in CVD. DSW has demonstrated its efficacy on lowering total cholesterol (TC) and LDL-C levels in hypercholesterolemic human subjects [14]. DSW administered in hypercholesterolemic rabbits exhibited lipid-lowering effects [17,18]. 

Epidemiological studies have demonstrated that the serum Mg^2+^ level is inversely correlated with the formation of atherosclerosis [19]. The Mg^2+^ supplement lowers the serum cholesterol and triglyceride levels and attenuates the atherosclerotic process in rabbits that are fed a high cholesterol diet [20,21]. These research studies indicated that dietary Mg^2+^ prevents the development of atherosclerosis in cholesterol-fed rabbits by inhibiting the lipid accumulation in the aortic wall [22]. Another study by Kishimoto *et al.* indicated that the Mg^2+^ supplement could inhibit fat absorption and improve postprandial hyperlipidemia in healthy subjects [23]. The co-administration of Ca^2+^ and Mg^2+^ significantly enhanced the cholesterol-lowering effects from plant sterols [24]. However, the Mg supplement appears to only produce a small, but clinically significant, reduction in blood pressure. A future prospective, large-scale, randomized trial should be conducted to further explore these results [25].

The purpose of the present study was to assess whether DSW from the Pacific Rim off of Hualien County could modulate systolic artery pressure (SAP) and diastolic artery pressure (DAP), reduce serum lipid levels, and prevent atherosclerosis formation. This study also investigated whether the molecular mechanisms underlying the lipid-lowering effects of DSW are associated with the AMPK-ACC pathway activation.

## 2. Results and Discussion

### 2.1. Body Weight Changes in Spontaneous Hypertensive Rats (SHRs)

Table 1 shows the changes in body weights of spontaneous hypertensive rats (SHRs) in the control, Lasix, 10% MgCl_2_, and DSW-treated groups. The body weights increased by approximately 189 g, 179 g, and 183 g after the administration with 0.1 × DSW, 1 × DSW, and 2 × DSW, respectively (equivalent to 3.75 mg/kg, 37.5 mg/kg, and 75 mg/kg Mg content). There were no signiﬁcant differences in body weights between the DSW-treated and the normal control groups after the four- and eight-week study.

**Table 1:** Changes in body weights of spontaneous hypertensive rats in the control, Lasix, 10% MgCl_2_, and deep sea water (DSW)-treated groups. * *p* < 0.05 compared to week 0.

Groups	Week 0 (g)	Week 2 (g)	Week 4 (g)	Week 8 (g)
Control	133 ± 10	189 ± 7 *	269 ± 9 *	300 ± 11 *
10 mg/mL, Lasix	131 ± 6	178 ± 12 *	261 ± 7 *	326 ± 5 *
10% MgCl_2_	122 ± 13	167 ± 8 *	255 ± 17 *	312 ± 22 *
0.1 × DSW (3.75 mg/kg/day)	131 ± 4	175 ± 9 *	260 ± 10 *	320 ± 14 *
1 × DSW (37.5 mg/kg/day)	133 ± 8	179 ± 18 *	264 ± 15 *	312 ± 13 *
2 × DSW (75 mg/kg/day)	130 ± 7	167 ± 4 *	255 ± 2 *	313 ± 8 *

### 2.2. DSW Lowers the Blood Pressures of Spontaneous Hypertensive Rats

Table 2, Table 3 show the SAP and DAP in the SHRs of control, Lasix, 10% MgCl_2_, and DSW-treated groups. The SAP and DAP were signiﬁcantly lower in the 1 × DSW- and 2 × DSW-treated groups than in the control group at the end of the four-week treatment. However, the SAP and DAP were signiﬁcantly lower even at the lowest concentration (0.1 × DSW) than in the control group in the eight-week study. Additionally, our results demonstrated that 10% MgCl_2_ significantly lowered the SAP and DAP in SHRs. Previous studies have indicated that feeding DSW pumped from Cape Muroto (Kochi Prefecture, Japan) demonstrated a preventive effect on mild hypertension in Kurosawa and Kusanagi-Hypercholesterolemic rabbits [15]. Our results, similarly, showed that DSW pumped from the Pacific Rim off the Hualien County (Hualien County, Taiwan) exhibited effects of lowering the SAP (Table 2) and DAP (Table 3) in SHRs. An earlier study indicated that Mg supplement might lower blood pressure by suppressing the adrenergic activity and, likely, natriuresis [26]. Another study showed that Mg^2+^ supplement corrected hypertension in mineralocorticoid-salt hypertensive animals by reducing the vascular tone [27].

**Table 2:** SAP changes in spontaneous hypertensive rats administered with Lasix, 10% MgCl_2_, and DSW-treated groups. * *p* < 0.05 compared to week 0; ^#^ *p* < 0.05 compared to the control group in the same time point.

Groups	Week 0 (mm Hg)	Week 2 (mm Hg)	Week 4 (mm Hg)	Week 8 (mm Hg)
Control	127 ± 6	164 ± 8 *	211 ± 8 *	244 ± 22 *
10 mg/mL, Lasix	134 ± 10	162 ± 17 *	193 ± 4 *	178 ± 14 *^,#^
10% MgCl_2_	135 ± 9	165 ± 11 *	181 ± 12 *^,#^	181 ± 3 *^,#^
0.1 × DSW (3.75 mg/kg/day)	131 ± 15	174 ± 7 *	226 ± 7 *	188 ± 12 *^,#^
1 × DSW (37.5 mg/kg/day)	125 ± 14	180 ± 8 *	176 ± 7 *^,#^	156 ± 16 *^,#^
2 × DSW (75 mg/kg/day)	126 ± 11	173 ± 7 *	162 ± 6 *^,#^	171 ± 18 *^,#^

**Table 3:** DAP changes in spontaneous hypertensive rats from treatments administered to the control, Lasix, 10% MgCl_2_, and DSW-treated groups. * *p* < 0.05 compared to week 0; ^#^ *p* < 0.05 compared to the control group in the same time point.

Groups	Week 0 (mm Hg)	Week 2 (mm Hg)	Week 4 (mm Hg)	Week 8 (mm Hg)
Control	81 ± 8	137 ± 9 *	150 ± 11 *	177 ± 19 *
10 mg/mL, Lasix	84 ± 12	138 ± 10 *	129 ± 5 *	129 ± 14 *^,#^
10% MgCl_2_	87 ± 11	115 ± 12 *	107 ± 13 *^,#^	124 ± 15 *^,#^
0.1 × DSW (3.75 mg/kg/day)	92 ± 12	132 ± 6 *	143 ± 10 *	121 ± 21 *^,#^
1 × DSW (37.5 mg/kg/day)	82 ± 6	128 ± 7 *	105 ± 5 *^,#^	112 ± 7 *^,#^
2 × DSW (75 mg/kg/day)	91 ± 9	134 ± 9 *	103 ± 7 *^,#^	120 ± 10 *^,#^

### 2.3. Body Weight Changes in New Zealand White Rabbits

Table 4 presents the changes in the body weights of rabbits in the control, 0.5% cholesterol, 0.01% lovastatin (Lova), 10% MgCl_2_, and DSW-treated groups. The body weights increased by approximately 0.97 kg, 0.82 kg, and 0.82 kg after the administration of 0.1 × DSW, 1 × DSW, and 2 × DSW, respectively. There were no signiﬁcant differences in the body weights between the DSW-treated and the normal control groups after eight-week studies.

**Table 4:** Changes in the body weights of rabbits in the control, 0.5% cholesterol, 0.01% lovastatin, 10% MgCl_2_, and DSW-treated groups.

Group	Control	0.5% Cholesterol	0.01% Lovastatin	10% MgCl_2_	0.1 × DSW	1 × DSW	2 × DSW
Initial weight (kg)	2.37 ± 0.54	2.47 ± 0.48	2.46 ± 0.50	2.65 ± 0.32	2.21 ± 0.65	2.17 ± 0.46	2.23 ± 0.41
Final weight (kg)	3.12 ± 0.33	3.19 ± 0.20	3.02 ± 0.34	3.23 ± 0.22	3.18 ± 0.48	2.99 ± 0.33	3.05 ± 0.25

### 2.4. Down-Regulatory Effect of Deep Sea Water (DSW) on Serum Total Cholesterol (TC)

**Figure 1:**
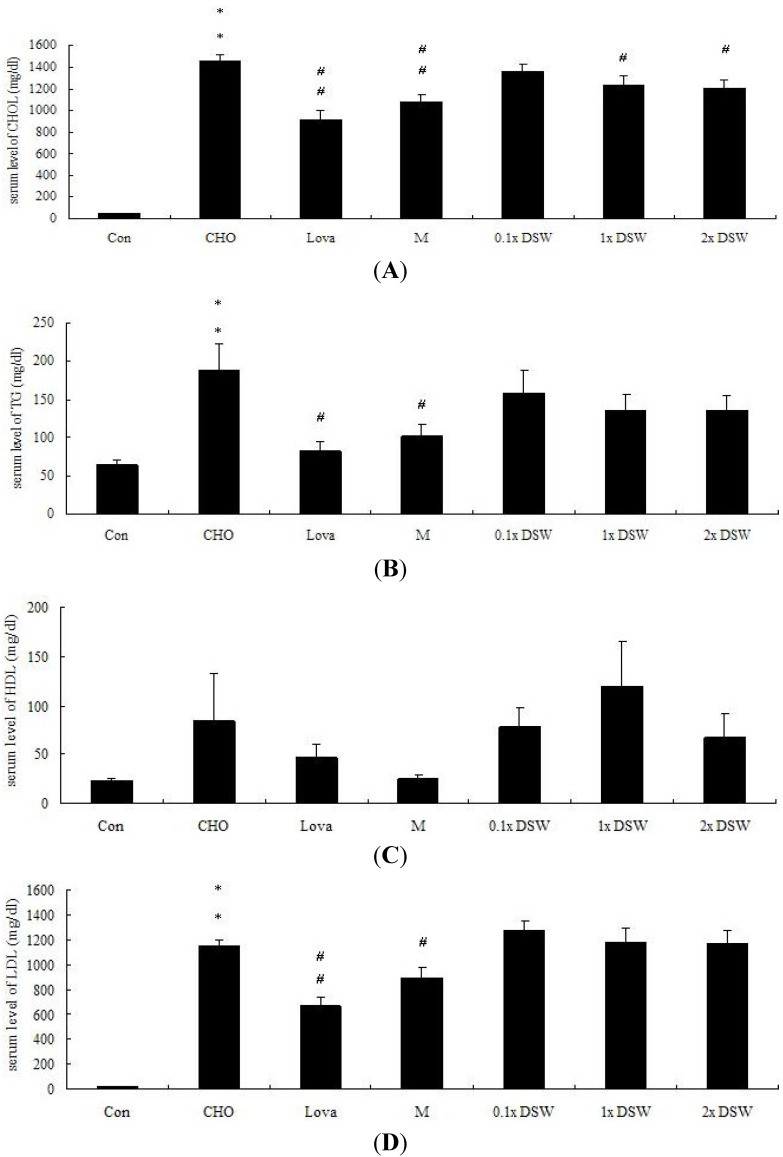
Serum chemical parameters were calculated in the high-fat-fed rabbit model (0.5% cholesterol) after an eight-week experiment. Control group (Con), 0.5% cholesterol diet (CHO), 0.5% cholesterol diet with 0.01% lovastatin (Lova), 0.5% cholesterol diet with a 10% MgCl_2_ (M), 0.5% cholesterol diet with 0.1 × DSW (0.1 × DSW), 0.5% cholesterol diet with 1 × DSW (1 × DSW), and 0.5% cholesterol diet with 2 × DSW (2 × DSW). CHOL, total cholesterol (**A**); TG, triglyceride (**B**); HDL, high-density lipoprotein (**C**); LDL, low-density lipoprotein (**D**). * *p* < 0.05; ** *p* < 0.01 compared to the control group; ^#^ *p* < 0.05; ^#^^#^ *p* < 0.01 compared to the cholesterol group.

The serum chemical parameters were examined to assess whether DSW could reduce the serum lipid profiles. Our results indicated that the plasma TC, triglyceride (TG), and LDL-C levels were improved after eight weeks of a 0.5% cholesterol diet (Figure 1a–c). In total, 1 × DSW, and 2 × DSW significantly improved the TC level by 1.18-, and 1.21-fold, respectively. The results have been consistent with findings in other studies [28,29], and the results were similar to the effects in the MgCl_2_ and lovastatin groups (Figure 1a).

Statins have been used in large randomized trials targeting lipid-lowering therapy and reduced risk of cardiovascular death, non-fatal MI, and stroke [30]. Statins inhibit HMGCR, thus reducing the production of cholesterol in the liver and up-regulating the LDL receptors to uptake LDL-C into the liver. Additionally, the Mg supplement decreases the levels of aortic cholesterol, and particularly cholesteryl ester in mice [31].

### 2.5. Down-Regulatory Effect of DSW on Fatty Liver Status and Lipid Accumulation

**Figure 2:**
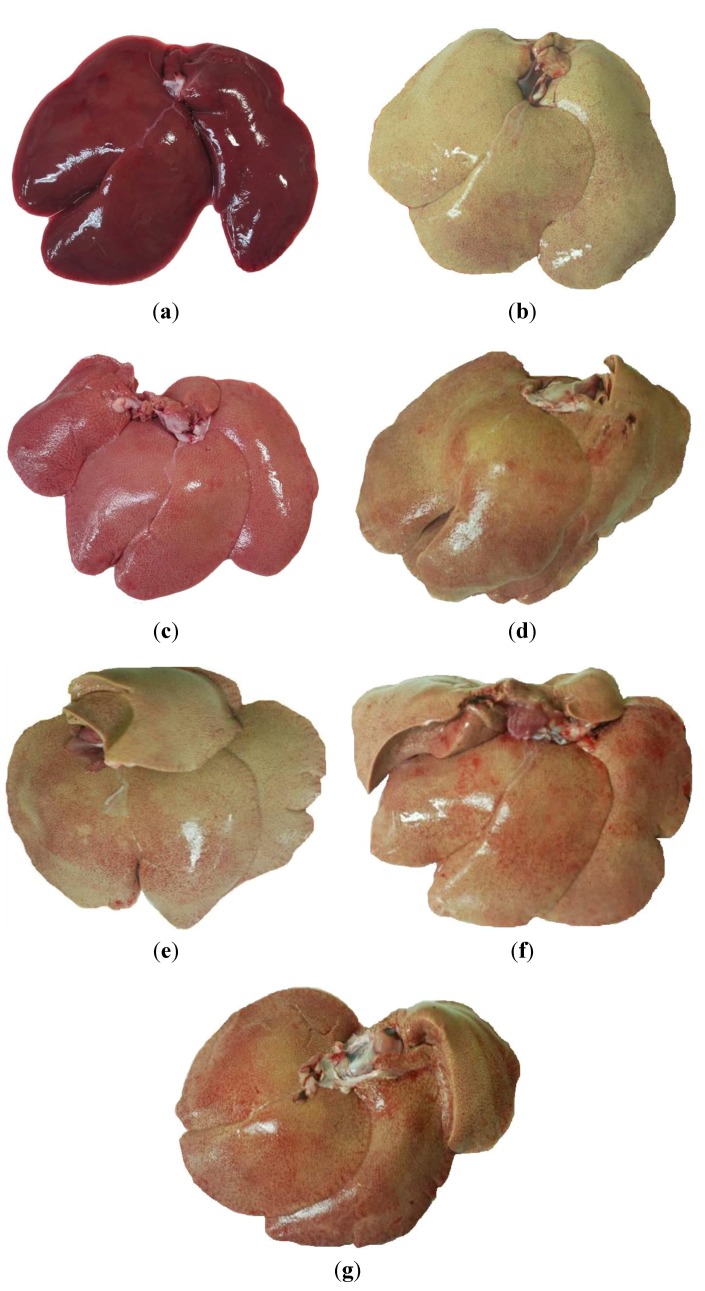
Photographs of liver appearance in the high-fat-fed rabbit model (0.5% cholesterol) after an eight-week study. (**a**) Control group; (**b**) 0.5% cholesterol diet; (**c**) 0.5% cholesterol diet with 0.01% lovastatin; (**d**) 0.5% cholesterol diet with a 10% MgCl_2_; (**e**) 0.5% cholesterol diet with 0.1 × DSW; (**f**) 0.5% cholesterol diet with 1 × DSW; and (**g**) 0.5% cholesterol diet with 2 × DSW.

**Figure 3:**
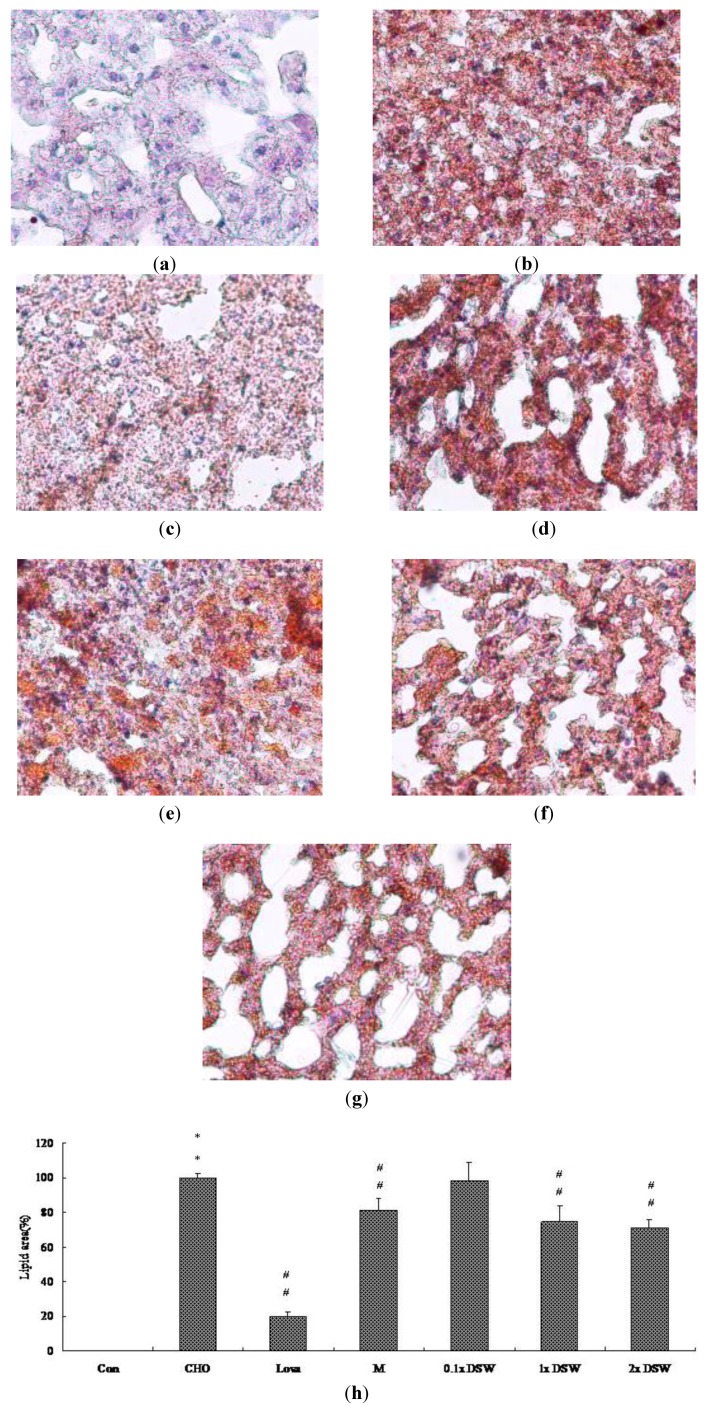
Histopathochemical examination of liver tissues in the hypercholesterolemic rabbit model after the eight-week study. (**a**) Control group (Con); (**b**) 0.5% cholesterol diet (CHO); (**c**) 0.5% cholesterol diet with 0.01% lovastatin (Lova); (**d**) 0.5% cholesterol diet with a 10% MgCl_2_ (M); (**e**) 0.5% cholesterol diet with 0.1 × DSW (0.1 × DSW); (**f**) 0.5% cholesterol diet with 1 × DSW (1 × DSW); (**g**) 0.5% cholesterol diet with 2 × DSW (2 × DSW); (**h**) densitometric analyses of (**a**–**g**). ** *p* < 0.01 compared to the control group; ^#^^#^ *p* < 0.01 compared to the cholesterol group.

A histopathological analysis of liver cryosections demonstrated that 0.5% cholesterol diet induced a phenomena-like fatty liver after eight-week administration (Figure 2b). Remarkably, 1 × DSW, and 2 × DSW reduced the severity of cholesterol diet-induced fatty liver (Figure 2f,g). This result is consistent with the result from the MgCl_2_ group (Figure 2d). A 0.5% cholesterol diet increased oil droplets accumulation within the liver tissue (Figure 3b); this effect was markedly reversed by 1 × DSW and 2 × DSW supplement (Figure 3f,g), which was similar to the results of the MgCl_2_ group (Figure 3d). 

### 2.6. Down-Regulatory Effect of DSW on Fatty Streak Lesions

**Figure 4:**
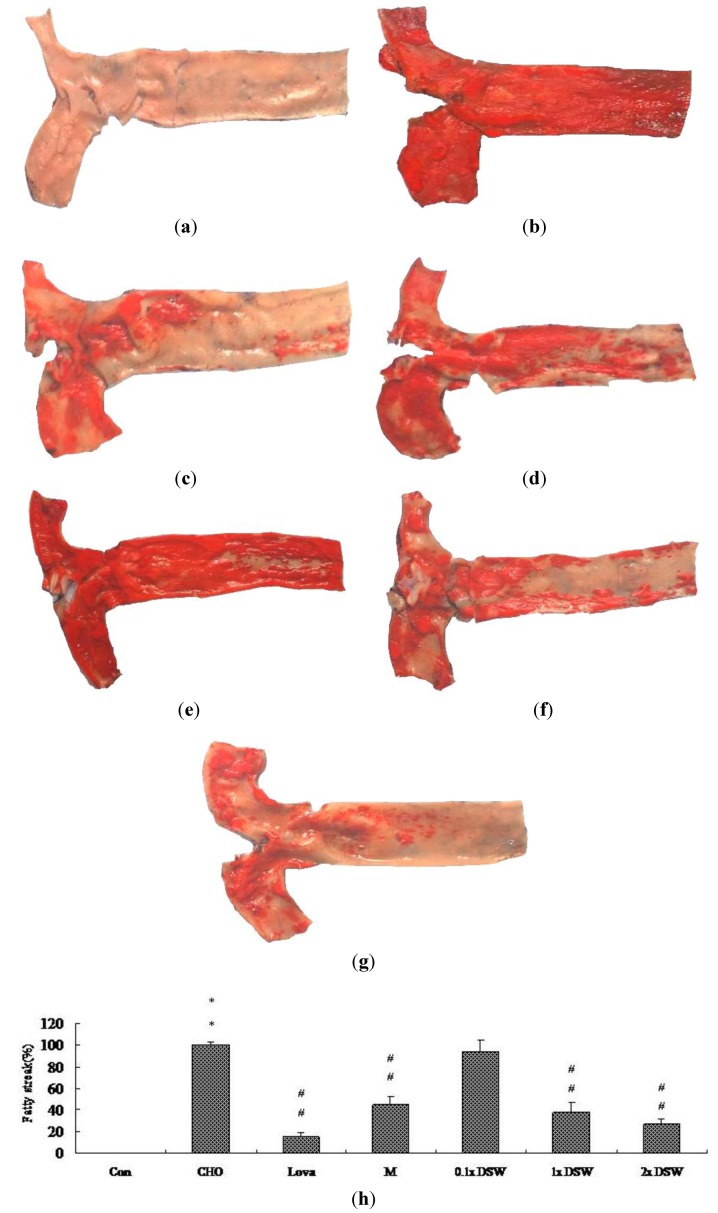
Histopathochemical examination of aortic fatty streak lesions in the hypercholesterolemic rabbit model after the eight-week study. (**a**) Control group (Con); (**b**) 0.5% cholesterol diet (CHO); (**c**) 0.5% cholesterol diet with 0.01% lovastatin (Lova); (**d**) 0.5% cholesterol diet with a 10% MgCl_2_ (M); (**e**) 0.5% cholesterol diet with 0.1 × DSW (0.1 × DSW); (**f**) 0.5% cholesterol diet with 1 × DSW (1 × DSW); (**g**) 0.5% cholesterol diet with 2 × DSW (2 × DSW); (**h**) densitometric analyses of (**a**–**g**). ** *p* < 0.01 compared to the control group; ^#^^#^ *p* < 0.01 compared to the cholesterol group.

Previously, Pan *et al.* reported the methods for the quantification of atherosclerotic lesions in the aortic root [29]. To determine the effect of DSW on the fatty streak formation, six-week-old male New Zealand white rabbits were fed with the 0.5% cholesterol diet for eight weeks. The fatty streak lesions stained with Sudan IV in the aorta root were used to determine if DSW could reduce the formation of atherosclerosis plaque (Figure 4). This study demonstrated that a 0.5% cholesterol diet considerably increased the aortic fatty streak lesions compared to the control group (Figure 4b). Additionally, 37.5 mg/kg and 75 mg/kg of DSW significantly reduced the intensity of the fatty streaks on the aorta intima compared to the CHO group (Figure 4f,g), which was similar to the MgCl_2_ group (Figure 4d). This study supports the hypothesis that inadequate intake of Mg results in an increase in atherosclerotic plaque development in rabbits [32].

### 2.7. Lipid-Modulating Effect of DSW and Lipid Metabolism-Associated Proteins

**Figure 5:**
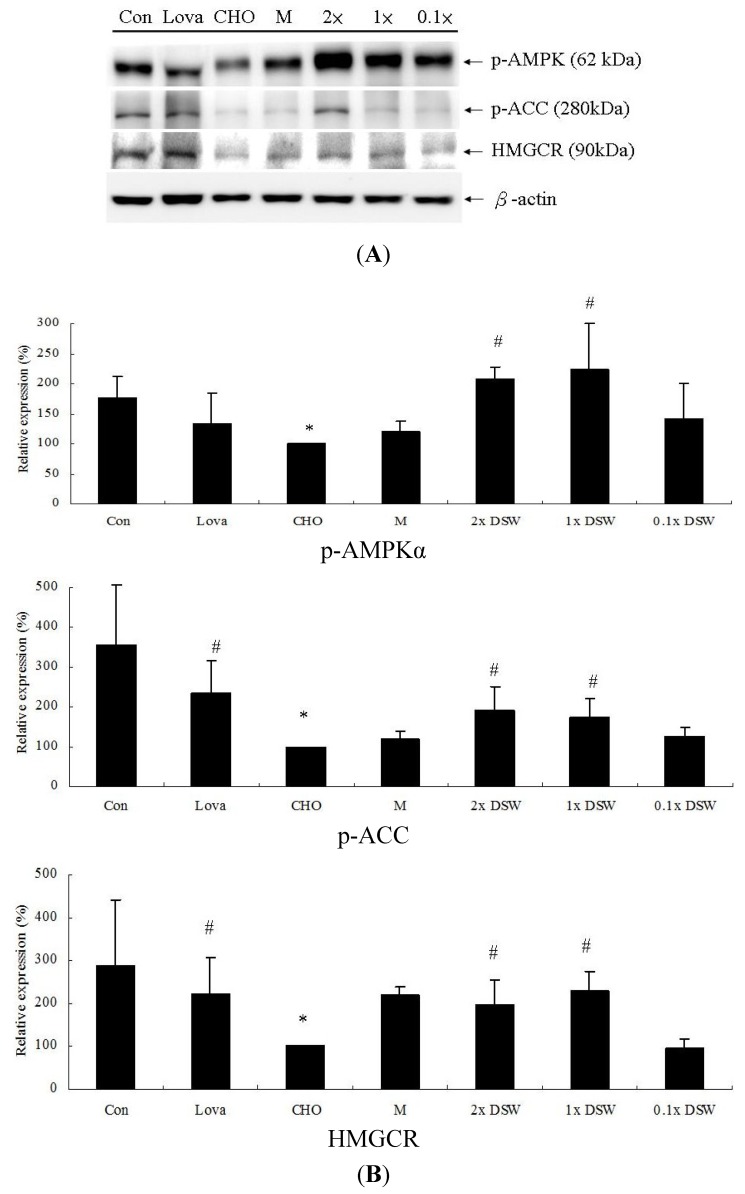
Protein expression of lipid metabolism associated molecules in the hypercholesterolemic rabbit model (*n =* 8 per group) after the eight-week study. (**A**) Control group (Con); 0.5% cholesterol diet (CHO); 0.5% cholesterol diet with 0.01% lovastatin (Lova); 0.5% cholesterol diet with a 10% MgCl_2_ (M); 0.5% cholesterol diet with 0.1 × DSW (0.1 × DSW); 0.5% cholesterol diet with 1 × DSW (1 × DSW); and 0.5% cholesterol diet with 2 × DSW (2 × DSW); (**B**) densitometric analyses of (**A**). * *p* < 0.05 compared to the control group; ^#^ *p* < 0.05 compared to the cholesterol group.

To determine how DSW affects the lipid-lowering effects, we investigated lipid metabolism-associated proteins, such as AMPK, ACC, and HMGCR. Our results demonstrated that the protein expression of AMPK phosphorylation, ACC phosphorylation, and HMGCR were significantly decreased after an eight-week treatment with the 0.5% (w/w) cholesterol diet (Figure 5). The addition of 1 × DSW and 2 × DSW returned the expression of these proteins to near basal levels compared to the cholesterol group, which showed a similar result as in the lovastatin group.

Our results first demonstrated that DSW might lower the lipid profiles in the rabbits via the AMPK-ACC pathways (Figure 5). AMPK plays an important role in lipid metabolism via the inhibition of HMGCR and ACC, thereby leading to fatty acid oxidation and inhibiting the production of cholesterol. Many studies have indicated that modulating the AMPK pathways significantly affects lipid metabolism. Platycodin D, saponins from the roots of *Platycodon grandiflorum*, increased the phosphorylation of AMPK and ACC in high-fat-diet-fed rats and activated AMPK via SIRT1/CaMKKβ in HepG2 cells, which was verified by the use of specific inhibitors [33]. Resveratrol has been proven to avoid liver fat accumulation induced by high-fat diet by increasing fatty acid oxidation and decreasing lipogenesis mediated via AMPK/SIRT1 signaling [34]. Si *et al.* demonstrated that *in vivo* administration of a novel synthesized indole compound improved the serum TG levels and decreased lipid accumulation in the livers of db/db mice [35]. Our results showed that DSW significantly stimulates AMPK and ACC phosphorylation (Figure 5B). An earlier study indicated that activated AMPK phosphorylate and consequently inactivated HMGCR [12]. However, our results showed that the total protein expression of HMGCR was upregulated by the DSW supplement (Figure 5B). We might speculate that these lower levels of sterols and non-sterol metabolites derived from mevalonate negatively regulated HMGCR. Additionally, HMGCR may be suppressed by cholesterol in the mammalian cells [36,37,38,39]. Therefore, this upregulated HMGCR protein, after an eight-week DSW supplement, might be initiated by their low levels of serum cholesterol (Figure 1a).

In addition to the beneficial effects of these minerals on the cardiovascular system, unknown effects of several ultratrace elements or unknown substances in DSW may be found in the future. Several inorganic trace substances, such as boron, rubidium, and vanadium, demonstrate higher concentrations in DSW. A boron-containing compound inhibits cholesterol biosynthesis by suppressing HMG-CoA reductase gene expression in hepatocytes [40].

## 3. Experimental Section

### 3.1. Materials and Production of the DSW

DSW was acquired from the Pacific Rim at a depth of 662 m, five kilometers off of Hualien County, Taiwan. We used DSW to conduct a series of procedures, including filtration, reverse osmosis, and concentration, as described by Fu *et al.* (2012) [14]. This concentrated DSW (deep ocean minerals; LC-90K) had a hardness of 400,000 mg/L, and the Mg content was 96,000 mg/L. The concentrated DSW from the deep ocean had been identified and compared with surface sea water (Table 5). The DSW used in this experiment was pasteurized, bottled, and provided by Taiwan Yes Deep Ocean Water Co., Ltd., Hualien, Taiwan.

**Table 5:** Mineral contents of surface sea water and DSW used in this study were analyzed by SGS Taiwan Ltd. LC-90K indicates liquid concentrate with content of Mg^2+^ over 90,000 ppm.

	Surface Sea Water (mg/L)	Deep Sea Water (DSW) LC-90K (mg/L)
Na	10,800	7240
K	392	10,400
Ca	411	39
Mg	1290	96,100
Sr	8.1	0.17
B	4.45	320
Fe	0.003	0.25
Li	0.17	11.7
Cu	0.0009	0.22
Co	0.0004	0.26
Mo	0.01	0.62
Ni	0.0066	0.11
Cr	0.0002	0.087
Rb	0.12	1.2
Si	2.9	0.5
V	0.002	1.2
F	13	21.8
Br	67.3	5400
I	0.064	5.5

The antibody against phosphor-ACC and phospho-AMPKα were obtained from Cell Signaling Technology, Inc., Beverly, MA, USA. The primary antibodies against β-actin and anti-HMGCR were purchased from Abcam, Cambridge, MA, USA, and Millipore/Upstate, Bedford, MA, USA, respectively. HRP-labeled secondary antibodies against mouse IgG and rabbit IgG were acquired from Santa Cruz Biotechnology, Santa Cruz, CA, USA. All other reagents were purchased from Sigma-Aldrich, St. Louis, MO, USA. 

### 3.2. Animal Experimental Design

All animal care was conducted according to the institutional animal ethical guidelines of the China Medical University. The animals were housed in cages and given *ad libitum* access to food and water and maintained on a 12-h light/dark cycle. 

#### 3.2.1. Measurements of Blood Pressure and Heart Rate in SHRs

Sixty spontaneous hypertensive rats (250–300 g) were purchased from BioLasco Taiwan Co., Ltd., Nankang, Taiwan. The animals sustained one week of adaptation; subsequently, they were randomly divided into six groups and fed the following diets for eight weeks until they were euthanized: A control group; 10 mg/mL of Lasix (Lasix group); 10% (w/w) MgCl_2_ supplement (MgCl_2_ group); 0.1 × DSW (0.1 × DSW group, equivalent to 3.75 mg/kg Mg); 1 × DSW (1 × DSW group, equivalent to 37.5 mg/kg Mg); and 2 × DSW (2 × DSW group, equivalent to 75 mg/kg Mg). All animals were kept in cages on a 12-h day/night cycle. The SBP, DBP, and HR were monitored in conscious animals by the tail-cuff method (MK-2000ST; Muromachi, Japan).

#### 3.2.2. Lipid-Lowering Effects

Fifty-six male New Zealand white rabbits (1500–2000 g) were purchased from the Animal Health Research Institute (Council of Agriculture, Executive Yuan, Tainan, Taiwan). Animals sustained one week of adaptation; subsequently, they were randomly divided into seven groups and fed the following diets for eight weeks: A regular diet (control group) (Fwu Sow Ind., Taichung, Taiwan); 0.5% cholesterol diet alone (CHO group); 0.5% cholesterol diet with 0.01% (w/w) lovastatin supplement (Lova group) (Yung Shin Pharm. Ind., Taipei, Taiwan); 0.5% cholesterol diet with a 10% (w/w) MgCl_2_ supplement (MgCl_2_ group); 0.5% cholesterol diet with 0.1 × DSW (0.1 × DSW group); 0.5% cholesterol diet with 1 × DSW (1 × DSW group); and 0.5% cholesterol diet with 2 × DSW (2 × DSW group). The daily feeding amount for each rabbit was 50 g/kg body weight per day. At the beginning and end of the eight-week study, the rabbits were anesthetized by an intramuscular injection of Zoletil 50^®^ (1 mL/kg) (Virbac Ltd., Carros, France), and the blood samples were harvested. Finally, the aortas (from the aortic arch to the bifurcation of the iliac arteries) and whole livers were collected from the rabbits after they were sacrificed for additional histopathological and western blotting assays.

### 3.3. Measurement of Serum Chemical Parameters

This study was based on our previous study [29]. The rabbits were fasted for 12 h; the blood samples were collected from the marginal ear veins of rabbits into BD Vacutainer™ EDTA blood collection tubes. The plasma was separated by centrifugation at 3000 rpm at 4 °C for 10 min. We measured the changes in blood chemistry parameters, including the serum levels of LDL, high-density lipoprotein (HDL), TC, TG, AST, and ALT (CheChang Co., Ltd., Taichung, Taiwan). 

### 3.4. Cryosectioning of Liver Tissues

Based on our previous study [29], we perfused the rabbit liver tissues with normal saline and fixed in 10% (v/v) formalin neutralized solution (J.T. Baker, Inc., Phillipsburg, NJ, USA) for 24 h. Subsequently, the tissues were embedded in Tissue-Tek^®^ OCT Compound (Sakura Finetek Inc., Torrance, CA, USA). The embedded tissues were cut into 10 μm-thick slices and stained with Sudan IV and hematoxylin (Merck, Darmstadt, Germany). The slices were washed with pure water for one minute to remove the OCT compound, washed with 50% (v/v) ethanol for 30 s, and then stained with 2% (w/v) Sudan IV for one hour. After additional washing with 50% (v/v) ethanol and pure water for two minutes, the slices were counterstained with hematoxylin. Photographs were obtained using a microscope equipped with a 10-fold magnification objective and quantified using an Alpha Imager 2200 documentation system (Alpha Innotech, Santa Clara, CA, USA). The manifestation of fatty liver progression was presented as the percentage of the area of oil droplets to the total liver tissues (cells).

### 3.5. Aortic Fatty Streak Staining

This study was based on our previous published research [29]. We opened the aortas longitudinally to expose the intimal surface and rinsed gently with normal saline. The aortas were incubated in 2% (w/v) Sudan IV, rinsed with several concentrations (100%, 90%, 80%, 70%, and 60%) of ethanol for one minute, and rinsed with pure water. The photographs were obtained using a digital camera (Nikon D80, Tokyo, Japan) and quantified using an Alpha Imager 2200^®^ documentation system (Alpha Innotech, Santa Clara, CA, USA). The progression of the fatty streak lesions was presented as the percentage of the stained area to the total area.

### 3.6. Western Blot

Based on our previous study [41], we extracted the proteins from the frozen liver tissues that were subjected to SDS-PAGE under reducing conditions on 10% acrylamide gels and transferred to polyvinylidene fluoride (PVDF) membranes by electroblotting. After a blockade of nonspecific binding sites, the membranes were incubated with primary antibodies (1:1000 dilution), followed by horseradish peroxidase-conjugated secondary antibodies (1:2000 dilution). The protein expression was visualized using SuperSignal^®^ West Pico Chemiluminescent Substrate (Thermo Scientific, Rockford, IL, USA), and the luminescence signal was acquired and analyzed using a Fujifilm LAS-4000^®^ system (Tokyo, Japan). The amount of p-AMPKα, p-ACC and HMGCR were expressed relative to the amount of β-actin.

### 3.7. Statistical Analysis

All values are expressed as the mean ± standard deviation (SD). The data were compared using a one-way analysis of variance (ANOVA) to evaluate the differences among multiple groups. *p* < 0.05 was considered to be statistically significant.

## 4. Conclusions

Our experimental study demonstrated that the DSW supplement from the Pacific Rim off of Hualien County (Taiwan) can attenuate mild hypertension (Table 2, Table 3), reduce serum TC (Figure 1), decrease lipid accumulation in tissues (Figure 2, Figure 3), and diminish aortic fatty streak lesions (Figure 4). Moreover, the lipid-lowering effects of the DSW may be partially mediated by the activation of AMPK/ACC molecular signaling (Figure 5). The liver damage, evidenced by the plasma levels of aspartate aminotransferase (AST) and alanine aminotransferase (ALT), was not noticeable with the DSW supplement at the end of an eight-week study (data not shown). The highest concentration (2 × DSW) treatment had no effects on the serum AST and ALT (data not shown). Our data showed that the 0.5% cholesterol diet led to a 4.22-fold increase in the tissue MDA content compared to the control group and that 1 × DSW and 2 × DSW supplements reduced the MDA content by 1.18- and 1.21-fold, respectively (data not shown).

These results suggest that DSW may have the potential to be developed as a hypotensive and lipid-lowering therapeutic agent or medicinal health food for the prevention or treatment of cardiovascular diseases, such as atherosclerosis.
